# Neuroprotective Effect of the Marine-Derived Compound 11-Dehydrosinulariolide through DJ-1-Related Pathway in In Vitro and In Vivo Models of Parkinson’s Disease

**DOI:** 10.3390/md14100187

**Published:** 2016-10-17

**Authors:** Chien-Wei Feng, Han-Chun Hung, Shi-Ying Huang, Chun-Hong Chen, Yun-Ru Chen, Chun-Yu Chen, San-Nan Yang, Hui-Min David Wang, Ping-Jyun Sung, Jyh-Horng Sheu, Kuan-Hao Tsui, Wu-Fu Chen, Zhi-Hong Wen

**Affiliations:** 1 Doctoral Degree Program in Marine Biotechnology, National Sun Yat-Sen University, 70 Lien-Hai Road, Kaohsiung 804, Taiwan; qscjuejuejue@gmail.com (C.-W.F.); hanchun25@gmail.com (H.-C.H.); anubis0620@gmail.com (C.-H.C.); sheu@mail.nsysu.edu.tw (J.-H.S.); 2 Doctoral Degree Program in Marine Biotechnology, Academia Sinica, 128 Academia Road, Section 2, Nankang, Taipei 115, Taiwan; 3 Marine Biomedical Laboratory & Center for Translational Biopharmaceuticals, Department of Marine Biotechnology and Resources, National Sun Yat-sen University, Kaohsiung 804, Taiwan; johnjohnkings@gmail.com; 4 College of Oceanology and Food Science, Quanzhou Normal University, Quanzhou 362000, China; davidw@dragon.nchu.edu.tw; 5 Genomics Research Center, Academia Sinica, Taipei 115, Taiwan; yrchen@gate.sinica.edu.tw (Y.-R.C.); biogoldfish@mail2000.com.tw (C.-Y.C.); 6 Department of Pediatrics, E-DA Hospital, School of Medicine, College of Medicine, I-SHOU University, Kaohsiung 840, Taiwan; y520729@gmail.com; 7 Graduate Institute of Biomedical Engineering, National Chung Hsing University, Taichung 402, Taiwan; 8 Center for Stem Cell Research, Kaohsiung Medical University, Kaohsiung 807, Taiwan; 9 Department of Medical Research, China Medical University Hospital, China Medical University, Taichung 404, Taiwan; 10 Graduate Institute of Marine Biotechnology, National Dong Hwa University, Pingtung 944, Taiwan; pjsung@nmmba.gov.tw; 11 Taiwan Coral Research Center, National Museum of Marine Biology & Aquarium, Pingtung 944, Taiwan; 12 Department of Marine Biotechnology and Resources, National Sun Yat-Sen University, 70 Lien-Hai Road, Kaohsiung 804, Taiwan; 13 Department of Obstetrics and Gynecology, Kaohsiung Veterans General Hospital, Kaohsiung 813, Taiwan; khtsui60@gmail.com; 14 Department of Obstetrics and Gynecology and Institute of Clinical Medicine, National Yang-Ming University, Taipei 112, Taiwan; 15 Department of Pharmacy and Graduate Institute of Pharmaceutical Technology, Tajen University, Pingtung County 907, Taiwan; 16 Department of Neurosurgery and Center for Parkinson Disease, Kaohsiung Chang Gung Memorial Hospital and Chang Gung University College of Medicine, Kaohsiung 833, Taiwan

**Keywords:** Parkinson’s disease, DJ-1, HO-1, CREB, zebrafish, neuroprotection

## Abstract

Parkinson’s disease (PD) is a neurodegenerative disorder characterized by tremor, rigidity, bradykinesia, and gait impairment. In a previous study, we found that the marine-derived compound 11-dehydrosinulariolide (11-de) upregulates the Akt/PI3K pathway to protect cells against 6-hydroxydopamine (6-OHDA)-mediated damage. In the present study, SH-SY5Y, zebrafish and rats were used to examine the therapeutic effect of 11-de. The results revealed the mechanism by which 11-de exerts its therapeutic effect: the compound increases cytosolic or mitochondrial DJ-1 expression, and then activates the downstream Akt/PI3K, p-CREB, and Nrf2/HO-1 pathways. Additionally, we found that 11-de could reverse the 6-OHDA-induced downregulation of total swimming distance in a zebrafish model of PD. Using a rat model of PD, we showed that a 6-OHDA-induced increase in the number of turns, and increased time spent by rats on the beam, could be reversed by 11-de treatment. Lastly, we showed that 6-OHDA-induced attenuation in tyrosine hydroxylase (TH), a dopaminergic neuronal marker, in zebrafish and rat models of PD could also be reversed by treatment with 11-de. Moreover, the patterns of DJ-1 expression observed in this study in the zebrafish and rat models of PD corroborated the trend noted in previous in vitro studies.

## 1. Introduction

There are currently two general goals in the pharmacological treatment of Parkinson’s disease (PD). The first approach intends to slow the loss of dopamine (DA), and the second approach attempts to improve the symptoms of PD. However, the current gold standard treatment for Parkinson’s disease is long-term treatment with Sinement^®^ (Kenilworth, NJ, USA). This treatment not only induces adverse events (including nausea, dizziness, confusion, insomnia, and cardiovascular changes) but also does not slow the progression of PD. Therefore, other preventative or therapeutic agents for the treatment of PD are urgently needed [1,2,3]. The compound 11-dehydrosinulariolide (11-de) is a cembranolide analogue that was isolated from the soft coral *Sinularia flexibilis* [4]. Lin et al. (2009) demonstrated that the colorless crystal has a dimension of 0.5 × 0.41 × 0.32 mm^3^. The crystalized compound was obtained by slow evaporation from a mixture of acetone/MeOH (1:1) solution. The chemical formula of 11-de is C_20_H_28_O_4_, and 11-de has an orthorhombic structure and a molecular weight of 332.42 g/mol [5]. Previous studies have demonstrated that 11-de has several bioactive properties, including anti-tumor, wound healing, and neuroprotective properties. Liu et al. (2011) found that 11-de inhibits cell proliferation in CAL-27 cells through glucose-regulated protein (GRP-78) and ubiquinol-cytochrome-c reductase complex core protein 1 (UQCRC-1) [6]. In addition, Su et al. (2012) revealed that 11-de induces apoptosis of A2058 cells through the dysregulation of mitochondria and the endoplasmic reticulum stress pathway [7]. Moreover, our previous study also indicated that 11-de protects neuronal-like cells against 6-OHDA-induced cytotoxicity through the PI3K/Akt pathway [8]. Liu et al. (2011) used two-dimensional gel electrophoresis (2-DE) to show that 11-de upregulated DJ-1 protein expression [6]. Several studies have reported that DJ-1 gene mutations can cause autosomal recessive PD [9,10,11].

DJ-1, also known as PARK7, was initially mapped as the third locus for the autosomal recessive, early-onset, inherited form of Parkinson’s disease [12]. It encodes peptides with 189 amino acids. There are several pathways, such as the nuclear factor erythroid-2 related factor 2 (Nrf2) pathway, the activation of which mediates a downstream anti-oxidative stress response, that may counter DJ-1-related oxidative stress, and prevent dopaminergic cell death in the substantia nigra (SN) [13]. Some studies indicate that treatment with DJ-1 recombinant protein, or by overexpression of DJ-1, may ameliorate 6-OHDA-induced PD symptoms in rat models of the disease [14,15]. DJ-1 may also enhance expression of p-Akt and its downstream antioxidative stress genes, namely Nrf2 [16]. Nrf2 is also a major transcription factor implicated in antioxidative stress and detoxification responses [17]. Upon exposure to oxidative stress, DJ-1 sequesters Kelch-like ECH-associated protein 1 (Keap1), leading to translocation of Nrf2 into the nucleus. Various antioxidative stress genes such as heme oxygenase-1 (HO-1) and superoxide dismutase (SOD) are activated, resulting in decreased levels of reactive oxygen species (ROS) [16]. Nrf2 also plays an important role in conferring neuroprotective effects in PD. Barone and Bohmann showed that upregulation of the Nrf2 pathway, or inhibition of its negative regulator, Keap1, could restore locomotor deficit in a fly model of PD [18]. Furthermore, the PI3K/Akt pathway also regulates the cAMP response element-binding protein (CREB) pathway. It has been found that Akt/protein kinase B (PKB) promotes cell survival by stimulating the expression of cellular genes via the CREB/CREB binding protein (CBP) nuclear transduction pathway. Previous studies have also demonstrated that the CREB/CBP pathway affects several populations of dopaminergic neurons [19,20]. To further clarify the role of 11-de in the DJ-1-mediated neuroprotective pathway, and its effect in in vivo models, we performed the experiments detailed below.

The mechanisms underlying the therapeutic effect of 11-de in PD remain ambiguous. Thus, our study investigated the mechanism of action of 11-de in SH-SY5Y cells, by looking at the DJ-1, p-Akt, Nrf2, and p-CREB pathways, and validating the therapeutic pathway with knockdown of DJ-1 expression. In addition, we attempted to further confirm the therapeutic effects of the compound in in vivo models by analyzing zebrafish larvae behavior. Finally, we detected variations in the expression of some biomarkers of PD, including tyrosine hydroxylase (TH) and DJ-1. In the future, we hope to promote this therapeutic compound, taking it from bench to bedside to help treat patients diagnosed with Parkinson’s disease.

## 2. Results

### 2.1. 11-de Enhanced 6-OHDA-Induced Upregulation of DJ-1 Expression

**Figure 1:**
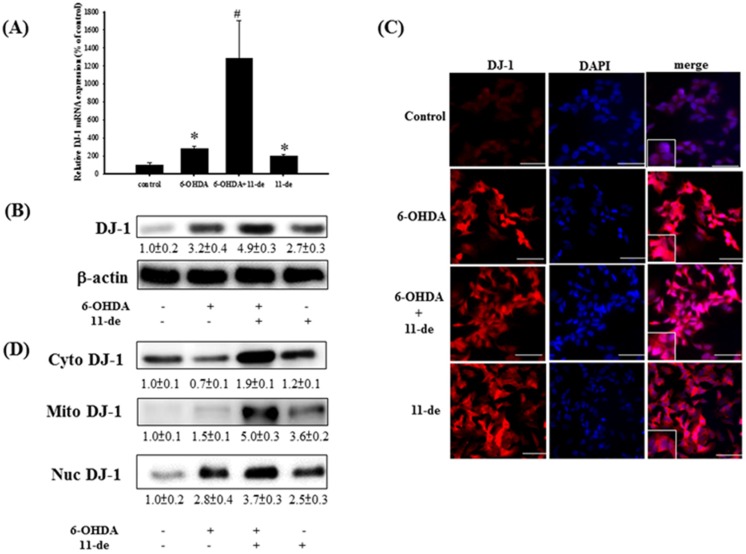
Effect of 11-de on DJ-1 protein expression and distribution in 6-OHDA treated SH-SY5Y cells. SH-SY5Y cells were pretreated with 10 nM 11-de for 1 h and incubated with 6-OHDA for 15 h. (**A**) Quantitative PCR of DJ-1 from the 6-OHDA-treated and 6-OHDA plus 11-de-treated groups; (**B**) western blotting of DJ-1 from the 6-OHDA-treated and 6-OHDA plus 11-de-treated groups. Treatment with 11-de significantly upregulated DJ-1 expression in protein and mRNA level (*n* = 3). SH-SY5Y cells were pretreated with 10 nM 11-de for 1 h and incubated with 6-OHDA plus 11-de for 15 h; (**C**) Immunofluorescence staining of DJ-1 (red) and DAPI (blue) in SH-SY5Y cells after pretreatment with 6-OHDA and 10 nM 11-de (scale bar = 50 μm) (*n* = 3). All cytoplasmic (cyto), nuclear (nuc), and mitochondrial (mito) fractions were obtained. SH-SY5Y cells were pretreated with 10 nM 11-de for 1 h and incubated with 6-OHDA for 15 h; (**D**) Western blotting of cytosolic, nuclear, and mitochondrial DJ-1 in control, 6-OHDA-treated, 6-OHDA plus 11-de-treated, and 11-de-treated groups (*n* = 3). Pretreatment with 10 nM 11-de for 1 h significantly enhanced 6-OHDA-induced upregulation DJ-1 expression in mitochondria and in nucleus. Each value represents the mean of three samples respectively, and error bars represent mean ± SEM. *, significantly different from the control group; #, significantly different from the 6-OHDA group (*p* < 0.05).

We examined the effect of 11-de on the expression of DJ-1. Pretreatment with 11-de (10 nM) for 1 h significantly enhanced the 6-OHDA-induced upregulation of DJ-1 mRNA and protein expression (Figure 1A,B, respectively). Double immunofluorescence staining (DJ-1 in red; the nucleus in blue) revealed that 6-OHDA, 6-OHDA + 11-de, or 11-de alone significantly upregulated DJ-1 protein expression in the cytosol and the nucleus compared to the control (Figure 1C). We further examined DJ-1 protein expression in the cytosol, nucleus, and mitochondria using western blotting. We found that 11-de enhanced 6-OHDA-induced upregulation of DJ-1 protein expression in the cytosol and nucleus. Notably, compared with the control, DJ-1 was upregulated in the mitochondria of 6-OHDA + 11-de and 11-de only treatment groups, but not the 6-OHDA treatment group.

### 2.2. Effect of 11-de on PTEN, Akt, HO-1, and Nrf2 Protein Expression, and Nrf2 Translocation in SH-SY5Y Cells

**Figure 2:**
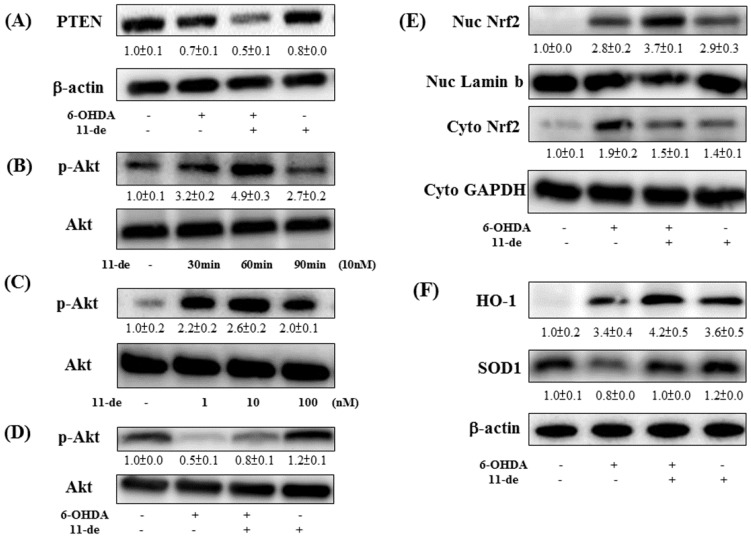
Effect of 11-de on PTEN, p-Akt, Nrf2, HO-1, and SOD-1 expression in SH-SY5Y cells. SH-SY5Y cells were pretreated with 11-de 10 nM for 1 h and incubated with 6-OHDA for 1 h. (**A**) Western blotting of PTEN from 6-OHDA-treated and 6-OHDA plus 11-de-treated groups (*n* = 3). Treatment with 10 nM 11-de significantly attenuated 6-OHDA-induced downregulation of PTEN expression in cells pretreated for 1 h, and in cells treated with 6-OHDA plus 11-de for 15 h. Cells were incubated with different concentrations of 11-de and for different periods of time. Western blotting of p-Akt expression (**B**) in groups treated with 11-de for 30 min, 60 min, and 90 min (*n* = 3). Treatment with 11-de significantly increased p-Akt expression in 30 min and 60 min treatment groups. Western blotting of p-Akt expression (**C**) in groups treated with 1 nM, 10 nM, and 100 nM 11-de for 60 min (*n* = 3). All three concentrations of 11-de significantly increased p-Akt expression. SH-SY5Y cells were pretreated with 11-de 10 nM for 1 h and incubated with 6-OHDA for 1 h. Western blotting of p-Akt (**D**) in control, 6-OHDA-treated, 6-OHDA plus 11-de-treated, and 11-de-treated groups (*n* = 3). Pretreatment with 10 nM 11-de for 1 h significantly reversed the 6-OHDA-induced downregulation of p-Akt. SH-SY5Y cells were pretreated with 11-de 10 nM for 1 h and incubated with 6-OHDA for 2 h. Western blotting of Nrf2 (**E**) in control, 6-OHDA-treated, 6-OHDA plus 11-de-treated, and 11-de-treated groups (*n* = 3). Pretreatment with 10 nM 11-de for 2 h significantly enhanced the 6-OHDA-induced nuclear translocation of activated Nrf2. The effect of 1 h treatment with 10 nM 11-de, and 16 h treatment of 6-OHDA plus 11-de on 6-OHDA-modulated SOD-1 and HO-1 expression in SH-SY5Y cells. Western blotting of SOD-1 and HO-1 (**F**) in control, 6-OHDA-treated, 6-OHDA plus 11-de-treated, and 11-de-treated groups (*n* = 3). Pretreatment with 10 nM 11-de for 15 h reversed 6-OHDA-induced downregulation of SOD-1 and enhanced 6-OHDA-induced upregulation of HO-1.

Pretreatment of SH-SY5Y cells with 10 nM 11-de for 1 h further downregulated 6-OHDA-induced downregulation of PTEN expression (Figure 2A). In addition, pretreatment with 11-de increased the expression of p-Akt at 30 min and 60 min, and p-Akt returned to baseline at 90 min (Figure 2B). Moreover, at concentrations ranging from 1 to 100 nM, 11-de pretreatment upregulated p-Akt expression at 60 min (Figure 2C). Pretreatment with 11-de (10 nM) significantly attenuated the 6-OHDA-induced downregulation of p-Akt expression (Figure 2D). Pretreatment with 11-de significantly enhanced 6-OHDA-induced upregulation of nuclear (Nuc) Nrf2 expression (Figure 2E). The downstream products in the Nrf2 pathway are SOD1 and HO-1. Our data show that 11-de significantly enhanced 6-OHDA-induced upregulation of HO-1, and attenuated the 6-OHDA-induced-downregulation of SOD-1 expression (Figure 2F). Moreover, pretreatment with 11-de alone significantly induced SOD-1 protein expression.

### 2.3. Effect of 11-de on 6-OHDA-Induced Upregulation of Phospho-cAMP Response Element-Binding Protein (p-CREB) and Brain-Derived Neurotrophic Factor (BDNF) Expression

**Figure 3:**
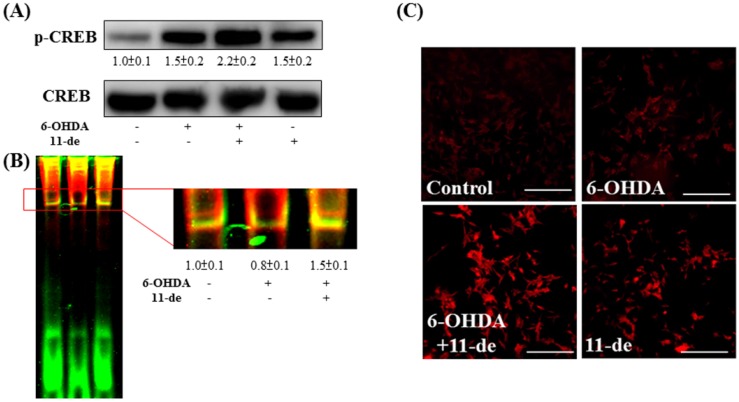
Effect of 11-de on p-CREB, and BDNF expression in 6-OHDA-treated SH-SY5Y cells. SH-SY5Y were pretreated with 10 nM 11-de for 1 h and 20 μM 6-OHDA plus 11-de for 3 h. Western blotting of p-CREB (**A**) in control, 6-OHDA-treated, 6-OHDA plus 11-de-treated, and 11-de-treated groups (*n* = 3). The expression of p-CREB was significantly upregulated after the pretreatment with 10 nM 11-de for 1h and 6-OHDA plus 11-de for 3 h in cells. Detection of the effect of 11-de on 6-OHDA-induced downregulation of CRE binding activity in SH-SY5Y cells in electrophoretic mobility shift assay (EMSA) analysis (**B**). The upper red rectangle indicates p-CREB, and the lower arrow indicates the free probe. EMSA of control, 6-OHDA-treated, and 6-OHDA plus 11-de-treated groups, respectively (*n* = 3). Treatment with 11-de significantly increased p-CREB expression in the SH-SY5Y cells pretreated with 10 nM 11-de for 1 h, and cells pretreated with 6-OHDA plus 11-de for 3 h. We further examined the effect of 11-de on brain-derived neurotrophic factor (BDNF) expression after 6-OHDA administration. SH-SY5Y cells were pretreated with 10 nM 11-de for 1 h and incubated with 6-OHDA for 15 h. (**C**) Immunofluorescence staining of BDNF (red) in the control, 20 μM 6-OHDA 15 h treatment, 6-OHDA plus 11-de-treated, and 11-de-treated groups (*n* = 3). Treatment with 11-de significantly upregulated BDNF expression both in the presence and in the absence of 6-OHDA administration.

We confirmed the effects of 11-de on p-CREB expression and the binding affinity of the CRE domain. Our result demonstrated that pretreatment with 11-de enhanced 6-OHDA-induced upregulation of p-CREB expression (Figure 3A). Moreover, 11-de significantly attenuated the 6-OHDA-induced downregulation of CBP binding affinity with the CRE domain, based on an electrophoretic mobility shift assay (EMSA) analysis (Figure 3B). We detected one of the downstream products of the CRE domain, namely, BDNF (Figure 3C). Pretreatment with 11-de upregulated the expression of BDNF in both the presence and absence of 6-OHDA. However, pretreatment with 6-OHDA alone did not alter BDNF expression.

### 2.4. The Effect of 11-de on p-Akt, HO-1, SOD-1, and Neuroprotective Activity in DJ-1-Knockdown Cells

**Figure 4:**
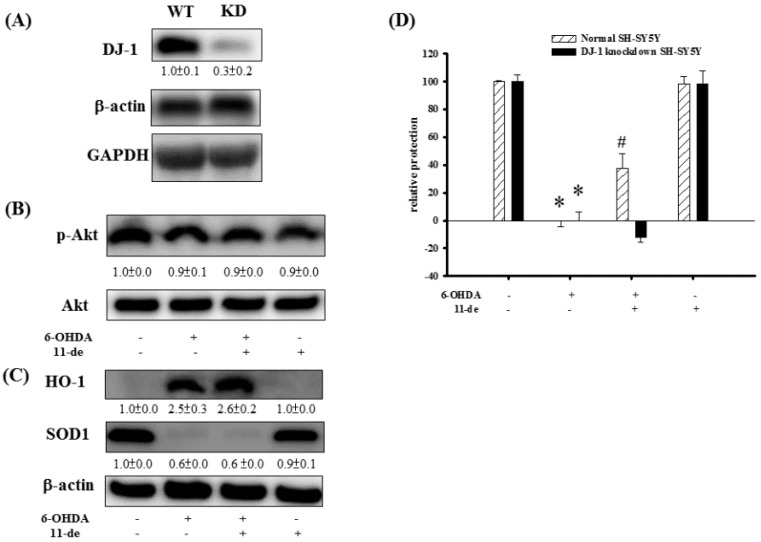
Effect of knockdown of DJ-1 expression in SH-SY5Y on 11-de neuroprotective activity, p-Akt, HO-1, and SOD-1 expression. SH-SY5Y cells treated with 100 nM DJ-1 siRNA for 24 h compared to untreated cells. (**A**) Western blot analysis of the DJ-1 protein in SH-SY5Y cells (*n* = 3). Results demonstrate that transfection of DJ-1 siRNA significantly downregulates expression of DJ-1. We examined p-Akt, HO-1, and SOD-1 expression in DJ-1 knockdown SH-SY5Y cells. The DJ-1 knockdown SH-SY5Y cells were pretreated with 10 nM 11-de and incubated with 6-OHDA for 1 h. Western blotting of p-Akt (**B**) in control, 6-OHDA-treated, 6-OHDA plus 11-de-treated, and 11-de-treated groups (*n* = 3). The upregulation effect of 11-de on p-Akt was inhibited by the knockdown of DJ-1. SH-SY5Y cells were pretreated with 10 nM 11-de for 1 h and 20 μM 6-OHDA plus 11-de for 15 h. Western blotting of SOD-1 and HO-1 (**C**) in control, 6-OHDA-treated, 6-OHDA plus 11-de-treated, and 11-de-treated groups in DJ-1 knockdown SH-SY5Y cells (*n* = 3). Knockdown of DJ-1 in SH-SY5Y cells inhibited the effect of 10 nM 11-de in reversing 6-OHDA-induced downregulation of SOD-1, and in attenuating 6-OHDA-induced upregulation of HO-1. Evaluation of DJ-1 knockdown SH-SY5Y relative protection following incubation with 6-OHDA plus 11-de. The normal SH-SY5Y cells were pretreated with 10 nM 11-de for 1 h and incubated with 6-OHDA for 18 h (*n* = 6). The Alamar blue assay results showed that pretreatment with 10 nM 11-de protected SH-SY5Y cells when incubated for 18 h with 20 μM 6-OHDA (**D**). The normal and DJ-1 knockdown SH-SY5Y cells were pretreated with 10 nM 11-de for 1 h and incubated with 6-OHDA for 18 h (*n* = 6). The Alamar blue assay results showed that pretreatment with 10 nM 11-de did not protect SH-SY5Y cells when incubated for 18 h with 20 μM 6-OHDA. The neuroprotective effect of 11-de was attenuated by the knockdown of DJ-1 expression. Each value represents the mean of three and six samples respectively, and error bars represent mean ± SEM. *, significantly different from the control group; #, significantly different from the 6-OHDA group (*p* < 0.05).

At 24 h post-siRNA transfection, DJ-1 expression levels were reduced to 34.4% (Figure 4A). There were no observable changes in p-Akt expression in 6-OHDA, 6-OHDA + 11-de, and 11-de only treatment groups in DJ-1-knockdown cells (Figure 4B). In addition, in DJ-1-knockdown cells, 11-de did not affect the 6-OHDA-induced upregulation of HO-1, or the downregulation of SOD-1 expression (Figure 4C). The relative protection percentage of the control, 6-OHDA-treated, 6-OHDA + 11-de (10 nM)-treated, and 11-de (10 nM)-treated groups in normal SH-SY5Y were 100% ± 0.9%, 0% ± 4.1%, 37.4% ± 10.7%, and 98.2% ± 5.7%, respectively. In DJ-1-knockdown SH-SY5Y cells, the relative protection percentage of the control, 6-OHDA-, 6-OHDA + 11-de-, and 11-de-only- treated groups were 100% ± 4.8%, 0% ± 6.4%, −11.9% ± 3.8%, and 98.5% ± 9.1%, respectively (Figure 4D). DJ-1-knockdown completely inhibited the protective effects of 11-de in 6-OHDA-treated SH-SY5Y cells.

### 2.5. The Protective Effects of 11-de on Locomotor Activity and Protein Expression in 6-OHDA-Treated Zebrafish

**Figure 5:**
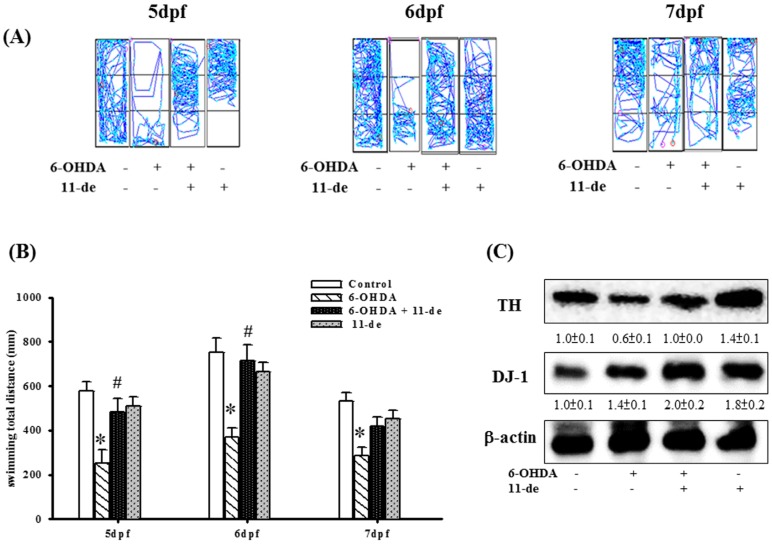
Effects of 11-de on 6-OHDA-induced locomotor deficit, downregulation of TH expression, and upregulation of DJ-1 expression. Zebrafish were treated with 0.1 μM 11-de (from 9 hpf (hour post fertilization) to 4 dpf) and with 250 μM 6-OHDA (from 2 to 4 dpf). (**A**) Typical swimming pattern at 5, 6, and 7 dpf in the control, 6-OHDA-treated, 6-OHDA plus 11-de-treated, and 11-de-treated groups (*n* = 16); (**B**) Total swimming distance at 5, 6, and 7 dpf for the control, 6-OHDA-treated, and 6-OHDA plus 11-de-treated groups (*n* = 16). Data shows that 11-de significantly reverses the 6-OHDA-induced deficiency of locomotor activity in 5, 6 dpf. Then, we detected the TH and DJ-1 expression. Zebrafish were treated with 0.1 μM 11-de (from 9 hpf to 4 dpf) and with 250 μM 6-OHDA (from 2 to 4 dpf); (**C**) Western blot analysis for TH and DJ-1 expression at 5 dpf in the control, 6-OHDA-treated, 6-OHDA plus 11-de-treated, and 11-de-treated groups (*n* = 3; twenty fish for each band). Our data shows that 11-de significantly reverses the decrease in expression of TH, a biomarker of dopaminergic neurons. Data shows that 11-de significantly increases the 6-OHDA-induced upregulation of DJ-1 expression. Each sample contained 20 zebrafish heads. Each value represents the mean of three samples, and error bars represent mean ± SEM. *, significantly different from the control group; #, significantly different from the 6-OHDA group (*p* < 0.05).

We established an in vivo zebrafish PD model in our previous study [21]. In the present study, we examined the therapeutic effects of 11-de in zebrafish. In the 6-OHDA-treated group, we found that total swimming distance was significantly inhibited at 5, 6, and 7 dpf (day post fertilization). Pretreatment with 11-de (0.1 μM) attenuated the 6-OHDA-induced downregulation of total swimming distance at 5 and 6 dpf (Figure 5A). Thus, 11-de significantly rescued the locomotor deficit that was induced by 6-OHDA in zebrafish (Figure 5B). However, 11-de did not significantly alter locomotor activity. We also assessed TH, an enzyme essential for dopamine synthesis and DJ-1 expression. Pretreatment with 0.1 μM of 11-de enhanced the 6-OHDA-induced upregulation of DJ-1 expression and attenuated the 6-OHDA-induced downregulation of TH expression. Moreover, in the group treated with only 11-de, the expression of DJ-1 was upregulated (Figure 5C).

### 2.6. Pretreatment with 11-de for 6-OHDA-Induced Lesions in a Rat Model of PD

**Figure 6:**
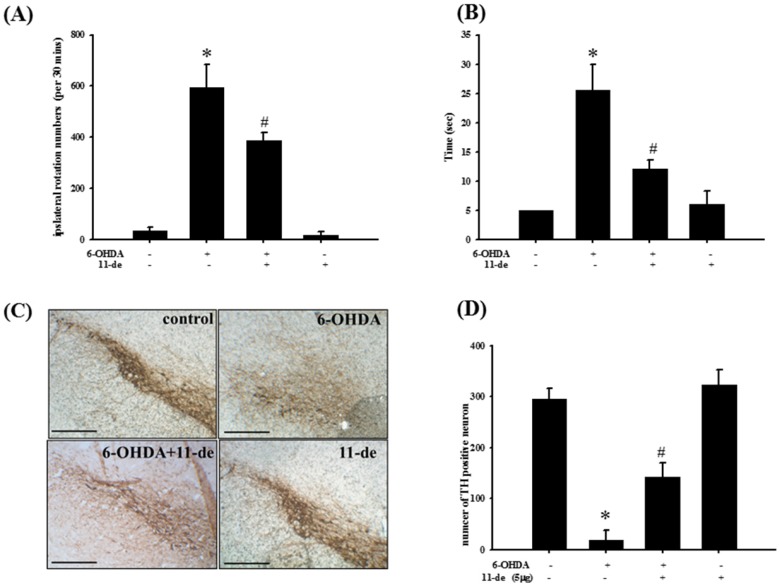
Treatment with 11-de protects against a methamphetamine-induced increase in the number of turns in rotation behavior, and an increase in time spent on the beam in the narrow beam test, and an increase in TH expression in 6-OHDA-induced rat model of PD. Rats were treated with 20 μg/kg 11-de (concentration 0.5 μg/μL for 10 μL) and 5 μg 6-OHDA plus 11-de. (**A**) Number of rotations analysis for rats at 14 days after lesion-induction in the sham, 6-OHDA-treated, 6-OHDA plus 11-de-treated, and 11-de-treated groups; (**B**) time spent on narrow beam for rats at 14 days after lesion in the sham, 6-OHDA-treated, 6-OHDA plus 11-de-treated, and 11-de-treated groups. Our data shows that 11-de significantly reverses the increase in number of turns in rotation behavior and reduces time spent on the beam in the narrow beam test. We also detected TH expression in the substantia nigra of 6-OHDA-induced rat models of PD. Rats were treated with 20 μg/kg 11-de (concentration 0.5 μg/μL for 10 μL) and 5 μg 6-OHDA plus 11-de. Whole-mount immunohistochemistry for TH expression at 14 days after lesions in (**C**) sham, 6-OHDA-treated, 6-OHDA plus 11-de-treated, and 11-de-treated groups; (**D**) quantitative results of TH-positive cells. Each bar was quantified by six samples. Our results demonstrated that 11-de significantly reverses 6-OHDA-induced downregulation of TH, which is a biomarker of dopaminergic neurons. Each value represents the mean of six samples, and error bars represent mean ± SEM. *, significantly different from the control group; #, significantly different from the 6-OHDA group (*p* < 0.05).

**Figure 7:**
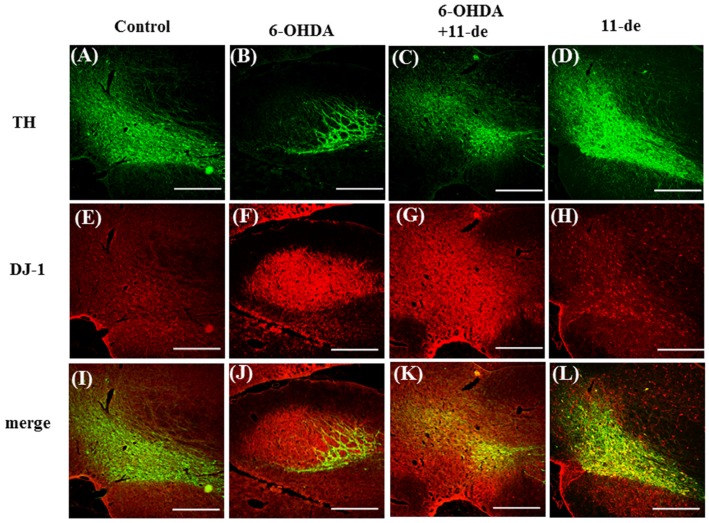
Effect of 11-dehydrosinulariolide on DJ-1 expression in a 6-OHDA-induced rat model of PD. Rats were treated with 5 μg/kg 11-dehydrosinulariolide (concentration 0.5 μg/μL for 10 μL), 20 μg 6-OHDA plus 11-dehydrosinulariolide and 11-dehydrosinulariolide alone for 14 days. The immunofluorescence staining of TH (green) (**A**–**D**) and DJ-1 (red) (**E**–**H**) in rat brains after 6-OHDA administration and 11-dehydrosinulariolide treatment, showing brains from the control (**I**); 6-OHDA-treated (**J**); 6-OHDA plus 11-dehydrosinulariolide-treated groups (**K**); and 11-dehydrosinulariolide alone (**L**) (*n* = 3). Treatment with 11-dehydrosinulariolide significantly increases expression of DJ-1 colocalized with TH, which is induced by 6-OHDA administration (scale bar = 100 μm).

To establish the neuroprotective effects of 11-de in an animal model, we tested this compound in a 6-OHDA-induced rat model of PD. The neurological function of PD-induced rats was evaluated based on the number of rotations and a narrow beam test. Intracerebroventricular injection of 11-de (5 μg) significantly attenuated the 6-OHDA-induced increase in the number of rotations (Figure 6A) and time spent on the narrow beams (Figure 6B). An immunohistochemical analysis was performed after two weeks of 6-OHDA administrations. The results show that 6-OHDA caused a 94.9% ± 6.23% loss of TH-immunoreactivity. In addition, 11-de significantly attenuated the 6-OHDA-induced downregulation of TH-immunoreactivity (Figure 6C). The number of TH-positive neurons in the control, 6-OHDA-, 6-OHDA + 11-de- and 11-de- treated groups was 296.3 ± 19.2, 19.0 ± 18.5, 143.3 ± 27.7, and 323.4 ± 29.6, respectively (Figure 6D). The fluorescence results showed that pretreatment with 11-de significantly attenuated the 6-OHDA-induced downregulation of TH (Figure 7A–D), and enhanced the 6-OHDA-induced upregulation of DJ-1 expression (Figure 7E–H). Moreover, double-immunostaining of the four treatment groups further confirmed that DJ-1 signals were mostly co-localized within dopamine neurons, especially in the 6-OHDA + 11-de and 11-de only treatment groups (Figure 7I–L).

## 3. Discussion

### 3.1. Summary

In our previous work, we only investigated 11-de protection of SH-SY5Y cells against 6-OHDA-induced damage via the PI3K/Akt pathway in an in vitro model [8]. However, our present study reveals that 11-de protects SH-SY5Y cell against 6-OHDA-induced cell damage via a DJ-1 modulated pathway that includes both the Akt/Nrf2 pathway and the Akt/CREB pathway. We also used siRNA to confirm the role of DJ-1 in the therapeutic mechanisms of 11-de activity. Moreover, we demonstrated that 11-de significantly attenuated 6-OHDA-induced damage in both zebrafish and rat models. As mentioned previously, DJ-1 has an important role in the therapeutic mechanism of 11-de. A previous study reported that the distribution of DJ-1 affects the antioxidant activity of 11-de [22].

### 3.2. Functions of DJ-1 in Different Locations

DJ-1 is mainly localized in the cytosol, but is also found in mitochondria and nuclei [23]. Previous research indicates that DJ-1 mediates different functions depending on its location. When located in the cytoplasm, DJ-1 modulates apoptosis signal-regulating kinase 1 (ASK1), protecting cells against apoptosis induced by TNF-α treatment [24,25]. Previous studies indicate that nuclear DJ-1 is increased in response to oxidative stressors to protect cells from death. Our findings reveal that treatment with 6-OHDA induces DJ-1 translocation into the nucleus. This finding is consistent with that of Kim et al. [26], who reported that DJ-1 translocates into the nucleus under conditions of oxidative stress. Mitochondrial DJ-1 appears to be primarily responsible for short-term protection against oxidative stress, demonstrating a greater protective effect than nuclear DJ-1, but via different mechanisms [23]. Corroborating the above findings, our results showed that treatment with 11-de enhanced the expression of mitochondrial DJ-1, protecting against 6-OHDA-induced damage. Our previous study showed that 11-de has anti-inflammatory properties, and that DJ-1 can also regulate inflammatory processes. DJ-1 directly interacts with Src homology region 2 domain-containing phosphatase-1 (SHP-1) and p-signal transducer and activator of transcription (STAT1). It may function as a scaffold protein, facilitating SHP-1 interactions with p-STAT1 and STAT1, thereby preventing extensive and prolonged STAT1 activation [27]. Our current results may explain our finding, as previously detailed [8]. We also showed that 11-de-mediated modulation of DJ-1/Akt could promote the downstream Nrf2/HO-1 and p-CREB/BDNF pathways. Other neuroprotective agents or clinical drugs may also affect these two pathways.

### 3.3. Effect of Neuroprotective Agents on Nrf2/HO-1 and CREB/BDNF Pathways

Deprenyl (phenyl-isopropyl-methyl-propargylamine, also known as Selegiline) is a selective monoamine oxidase-B (MAO-B) inhibitor used clinically in the treatment of PD [28]. Earlier research has indicated that Deprenyl affects neuroprotective activity by modulating the Nrf2/HO-1 pathway [29,30]. In Xiao et al., treatment with 10–100 μM Deprenyl activated the Nrf2 pathway by upregulating PI3K and MAPK pathways in the PC12 cell line. It also established the mechanism of action by inhibiting activity of Nrf2 [29]. Furthermore, Nakaso et al. revealed that Deprenyl exerted a neuroprotective activity in the SH-SY5Y cell line by activating the Nrf2/HO-1 pathway [30]. They also tested this drug in a rat model of PD. Pretreatment with Deprenyl significantly reversed the MPTP-induced locomotor deficit and attenuation of TH-expression [31,32]. Our results also indicate that, similarly to Deprenyl, 11-de may amplify the effects of the Nrf2/HO-1 pathway via activation of p-Akt. Some neuroprotective substances, such as curcumin and epigallocatechin gallate (EGCG), upregulate the CREB/BDNF pathway, similar to 11-de [33,34]. Curcumin is a major constituent of curcuma longa, a traditional Chinese medicine that is used to treat mental disorders. Previous studies have demonstrated that curcumin has neuroprotective effects in both in vitro and in vivo PD models [25,35]. Zbarsky et al. reported that treatment with curcumin significantly attenuated locomotor deficits and the downregulation of TH that was induced by 6-OHDA [25]. Furthermore, curcumin may increase p-CREB and BDNF expression [34]. In addition to curcumin, EGCG is the most abundant catechin in green tea, and is a polyphenol that is widely investigated for its potential benefit in many diseases. EGCG enhanced p-CREB and BDNF expression in in vitro models of PD [25]. Similar to curcumin and EGCG, 11-de also exerts neuroprotective activity via the p-CREB/BDNF pathway. Moreover, we found evidence concerning the relationship between DJ-1 and 11-de. We performed gene signature analysis to identify the effect of 11-de in 6-OHDA-treated SH-SY5Y cells (data not shown). The data demonstrated that the neuroprotective activity of 11-de was related to phosphodiesterase activity. DJ-1 also played an important role in regulating phosphodiesterase activity [36]. Our gene signature data revealed that 11-de demonstrated an antioxidative property similar to that of the clinical drug, Butein (data not shown). This drug’s antioxidative activity is achieved via regulation of Nrf2 translocation, and by increasing HO-1 expression [37]. These findings support the hypothesis that DJ-1 plays an important role in the mechanisms underlying the neuroprotective effects of 11-de.

### 3.4. Future Studies Involving 11-Dehydrosinulariolide

Nowadays, PD drugs focus on the replenishment of dopamine with alternative dopamine agonists, or on slowing the metabolism of the residual endogenous dopamine. However, thus far, no study has directly examined the ability of compounds to regulate activity of DJ-1. Therefore, we will further investigate the effect of 11-de treatment on the role of DJ-1 in zebrafish and rat models of PD. In addition, we intend to focus on changes upstream factors of DJ-1 to clarify the mechanism of 11-de. We will also attempt to develop this compound in different formulations, such as oral or intraperitoneal (i.p.) injection, to further conform to clinical requirements.

## 4. Materials and Methods

### 4.1. Cell Culture

The SH-SY5Y neuroblastoma cell line was obtained from the American Tissue Culture Collection (Rockville, MD, USA). The cells were grown at 37 °C in a 1:1 Dulbecco’s Modified Eagle’s Medium (DMEM, Invitrogen/Gibco, Carlsbad, CA, USA.) and Hams F12 Nutrient Mixture (Invitrogen/Gibco), containing 10% heat-inactivated fetal bovine serum (FBS).

### 4.2. Preparation of Nuclear Extracts

The extraction and isolation of nuclear fractions were performed with the ProteoJET™ Cytoplasmic and Nuclear Protein Extraction Kit (Fermentas, Vilnius, Lithuania), according to the manufacturer’s instructions. The procedure is based on cell lysis with mild detergents. The prepared cell extracts were compatible with western blotting and electrophoretic mobility shift assay (EMSA).

### 4.3. Electrophoretic Mobility Shift Assay (EMSA)

The sequences of double-stranded oligonucleotides used in EMSAs were CRE consensus, 5′-AGA GAT TGC CTG ACG TCA GAG AGC TAG-3′ (sc-2504, Santa Cruz, CA, USA). Nuclear extracts (10 μg) were incubated for 20 min at room temperature (RT) with the labeled oligonucleotide (1 × 10^5^ c.p.m.) in band shift buffer (25 mM Hepes, pH 7.9, 40 mM KCl, 3 mM MgCl_2_, 0.1 mM EDTA, 1 mM dithiothreitol, and 10% glycerol), containing 1 μg poly (dI-dC) as a non-specific competitor. DNA–protein complexes were resolved by electrophoresis on non-denaturing 6% polyacrylamide gels for 2–3 h at 150 V (50 mM Tris–HCl, 45 mM boric acid, 0.5 mM EDTA). The labeled oligonucleotide was purchased from commercialized product (sc-2504) and we use two fluorescent dyes, SYBR^®^ Green EMSA for RNA or DNA detection and SYPRO^®^ Ruby EMSA protein gel stain for protein detection (E33075, Thermo Fisher, Waltham, MA, USA). They were analyzed with the UVP BioChemi Imaging System, and relative densitometric quantification was performed using LabWorks 4.0 software (UVP, Upland, CA, USA).

### 4.4. Preparation of Mitochondrial Extracts

Mitochondrial proteins were isolated using a commercially available mitochondria isolation kit (BosterBio AR0156, Pleasanton, CA, USA). The protein concentration of mitochondrial pellets was determined as previously described [8]. The prepared cell extracts were compatible with western blotting.

### 4.5. Western Blotting

Western blotting was carried out as described previously [38,39]. In brief, an equal volume of sample buffer was added to the sample. Samples were loaded onto a 10% SDS-polyacrylamide gel and electrophoresed at 150 V for 60 min. After electrophoresis, the proteins were transferred overnight at 4 °C, using transfer buffer, onto a polyvinylidene difluoride (PVDF) membrane at 125 mA. The membranes were blocked for 50 min at RT with 5% non-fat dry milk and 0.1% TTBS, and incubated with the primary antibodies for 16 h at 4 °C. The membrane was washed three times in TTBS for 10 min, blocked with 5% non-fat dry milk/TTBS, and incubated for 1 h at RT with the secondary antibody, horseradish peroxidase (HRP)-conjugated anti-rabbit antiserum. The immune reactive bands were visualized by enhanced chemiluminescence. The images were visualized using the UVP BioChemi Imaging System, and relative densitometric quantification was performed using LabWorks 4.0 software.

### 4.6. Quantitative Real-Time PCR

Total RNA was extracted from SH-SY5Y of each treatment group using the TRIzol_Reagent (Invitrogen TM, Carlsbad, CA, USA) according to the manufacturer’s instructions. RNA was reverse transcribed to single-stranded cDNA using the iScript Cdna synthesis kit (Bio-Rad CFX manager, Hercules, CA, USA). We then performed real-time PCR using the iQTM SYBR_Green (Bio-Rad) supermix (Hercules, CA, USA) for human DJ-1 in the Bio-Rad real-time PCR system. Primers of DJ-1 were forward: 5′-GCCTGATTCTTACAAGCCGG-3′ and reverse: 5′-CAAGCGCAAACTCGAAGCT-3′. The expression level of each gene was expressed as a relative fold change (log 2 ratio) that was calculated using the comparative Ct method and using GAPDH as the internal reference.

### 4.7. Transfection of DJ-1 siRNA

The cells were transiently transfected with specific PARK7/DJ-1 siRNA (Life Technologies, Grand Island, NY, USA). The transient transfections with siRNAs were performed with LipofectAMINE 2000 PlusReagent (Invitrogen, Carlsbad, CA, USA). Three hours after transfection, the cultures were washed once with PBS and incubated with DMEM overnight. The DJ-1-knockdown cells were aliquoted to 6-cm dishes or 96 micro-well plates for further experimentation.

### 4.8. Zebrafish and Locomotor Test

The AB strain (wild type) of wild-type zebrafish was used for this study. Embryos were collected after natural spawning, staged according to standard criteria, and raised synchronously at 28.5 °C in Hank’s buffer. The locomotor behavior test of zebrafish larvae was performed as per the details in a previous study [21].

### 4.9. Ethical Approval

All animal care and experimental use of animals conformed to the Guiding Principles in the Care and Use of Animals of the American Physiology Society and was approved by the National Sun Yat-sen University Animal Care and Use Committee. All studies involving animals are reported in accordance with the ARRIVE guidelines for reporting experiments involving animals. Every effort was made to minimize both the number of animals used and their suffering. Animal use procedures were conducted in strict accordance with the *NIH Guide for the Care and Use of Laboratory Animals* (8th edition, 2011) [40]. Each rat was used only once during the study.

### 4.10. Animals

Twenty male Wistar rats (BioLASCO Co. Ltd., Taipei, Taiwan) at eight weeks old, weighing 260–285 g, were used in the experiments. The 20 rats were randomly divided into four groups: control, 6-OHDA treatment, 6-OHDA plus 11-de, and 11-de alone group (five animals in each group). All studies were reported in accordance with the ARRIVE guidelines for experiments involving animals [41,42]. The rats were maintained in Plexiglas cages in a temperature-controlled (22 °C) room, under a 12-h light/dark cycle, and given free access to food and water.

### 4.11. 6-OHDA-Induced Parkinson’s Study in Rats

The rats were anesthetized with isoflurane, and secured in a Kopf stereotactic instrument with the tooth bar set 15 mm above the interaural line. The animals were anesthetized with 2.5% isoflourane and treated concomitantly with either 20 μg/kg (concentration 0.5 μg/μL for 10 μL) 11-dehydrosinulariolide or vehicle (0.1% ascorbic acid in 0.9% saline), and 5 μg 6-OHDA. Unilateral injections of all solutions were administered into the left SN at the following coordinates: AP, −1.2; ML, −2.4; DV, −8.5 mm from the bregma. A 27-gauge Hamilton syringe connected to an infusion mini-pump was used to administer 5 μg 6-OHDA and 5 μg of 11-dehydrosinulariolide were delivered by the minipump at 5 μL/min. The syringe was left in place for 5 min before slowly retracting it to allow for toxin fusion, and prevention of reflux. Narrow beam tests and rotation behavior were modified from procedures previously described [43,44,45,46]. In brief, two weeks after intra-nigral stereotaxic injection of 6-OHDA, animals were subjected to rotational behavior testing. Rats were injected subcutaneously (s.c.) with 5 mg/kg d-amphetamine, placed in a cylindrical cage (240 mm diameter, 300 mm height), and the number of rotations, both ipsilateral and contralateral, completed during a 30-min period were recorded using a digital camera and counted. The narrow beam used for the experiments was wooden, 200 cm long, 4 cm wide, and 3 cm tall. The beam was suspended 100 cm from the ground by wooden supports at either end. The wooden supports formed a sheer drop at the “start” of the beam; a platform was located at the “end,” next to which the home cage of the test rat was placed. The stopwatch was stopped when all four of the rat’s feet were placed upon the finishing platform at the end of the beam. Rats were sacrificed by perfusion with ice-cold PBS and 4% paraformaldehyde two weeks after lesion. For all the behavior described above, the measurements were made in a blinded fashion, meaning that the surgery operator only marked the number on the tail and the measurements were made by other analysts.

### 4.12. Immunohistochemistry

Cultures and tissues were fixed with 4% paraformaldehyde for 10 min at RT. Cultures were incubated in blocking buffer for 1 h, then in a 1 μg/mL dilution of anti-Nrf2, anti-DJ-1, anti-BDNF or anti-TH antibody for 2 h, 2 h, 2 h, and 15 h, respectively. The culture placed in secondary antibody (Alexa 568 anti-rabbit IgG, 1 μg/mL, Molecular Probes, Eugene, OR, USA) for 2 h at RT. Cultures were rinsed again with TBST as before, mounted on glass slides (Fisher Scientific, Waltham, MA, USA) with SlowFade Light (Molecular Probes, Eugene, OR, USA), and visualized using Leica Optical’s Confocal Laser Scanning Microscopy System (Leica, DM6000B) with the SPOT program (Diagnostic, RT slider SPOT, Hamilton, CA, USA).

### 4.13. Statistical Analysis

All data are represented as the mean ± SEM. For immunoreactivity data, the intensity of each test band was expressed as the relative optical density (OD) calculated from the average control OD values, as obtained from all controls. Wherever applicable, data were analyzed using one-way analysis of variance (ANOVA) followed by Dunnett’s test when found to be significant. A *p* value of less than 0.05 was considered statistically significant. Sigmastat was used for statistical analysis of the data.

### 4.14. Chemical and Antibodies

1. 6-OHDA (6-hydroxydopamine, Sigma, St. Louis, MO, USA; catalog No. H4381)
2. β-actin (a loading control; dilution, 1:1000) (Sigma, St. Louis, MO, USA; catalog No. A5441)
3. BDNF (brain derived neurotrophic factor, dilution 1:100) (Millipore, Billerica, MA, USA; catalog No. AB1779SP)
4. HO-1 (heme oxygenase-1, dilution, 1:1000) (Cell Signaling Technology, Danvers, MA, USA; catalog No. 5061)
5. Nrf2 (dilution, 1:1000) (Abcam, biorbyt, Cambridge, UK; catalog No. ab31163)
6. PARK7/DJ-1 (dilution, 1:1000) (Abcam, biorbyt, Cambridge, UK; catalog No. ab18257)
7. p-Akt (Protein kinase B; dilution, 1:1000) (Cell Signaling Technology, Danvers, MA, USA; catalog No. 9271)
8. p-CREB (cAMP response element binding protein, dilution, 1:1000) (Santa Cruz Biotechnology Inc., Dallas, TX, USA; catalog No. sc-7978)
9. TH (tyrosine hydroxylase; dilution 1:1000) (Millipore, Billerica, MA, USA; catalog No. MAB318)

## 5. Conclusions

**Figure 8:**
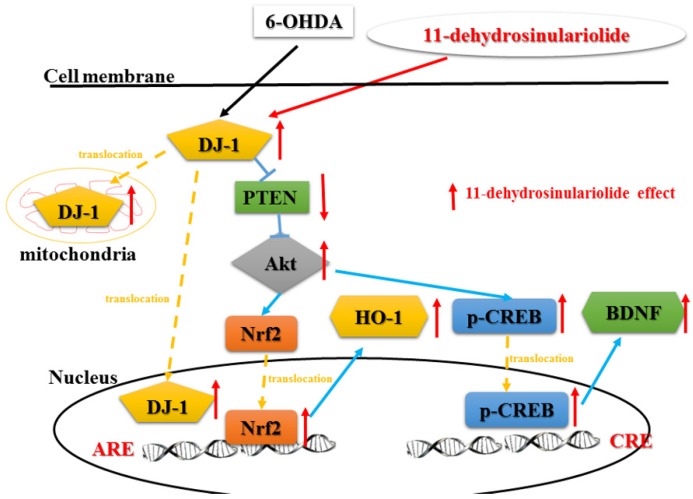
Putative therapeutic mechanisms of action of 11-de in 6-OHDA-induced cell death. 6-OHDA increases the upregulation of DJ-1 protein expression and translocates from the cytosol into the nucleus and mitochondria. The cytosolic DJ-1 inhibits downstream signaling via PTEN. The 6-OHDA-induced downregulation of PTEN activates the Akt pathway and its downstream Nrf2 and p-CREB pathways. Activation of Akt induces Nrf2 translocation into the nucleus, and results in the upregulation of HO-1, but not SOD-1. Moreover, 6-OHDA increases the translocation of p-CREB into nucleus and the upregulation of BDNF expression. The compound 11-de not only significantly enhanced 6-OHDA-induced upregulation of cytosolic DJ-1 expression but also promoted DJ-1 translocation into mitochondria and the nucleus. In contrast, 11-de activates Akt and induces the upregulation of Nrf2 and p-CREB. The 11-de-induced upregulation of DJ-1 inhibits downstream PTEN and attenuates the 6-OHDA-induced downregulation of Akt. The translocation of Nrf2 into the nucleus leads to the upregulation of HO-1 and SOD-1 expression. In contrast, 11-de also increases the expression of p-CREB, and increases its binding affinity with the CRE domain. In addition, 11-de upregulates expression that is downstream of the p-CREB pathway, such as BDNF expression.

Our results show that 11-de may regulate expression of DJ-1, and activate the Akt/PI3K, p-CREB, and Nrf2/HO-1 pathways (Figure 8). In addition, we found that 11-de could reverse the 6-OHDA-induced increase in total swimming distance in a zebrafish model of PD. Treatment with 11-de reversed the 6-OHDA-induced increase in the number of turns and the time spent on the beam in a rat model of PD. We showed that 11-de reverse 6-OHDA-induced downregulation of TH in both zebrafish and rat models of PD.

## References

[1] Thanvi B.R., Lo T.C. (2004). Long term motor complications of levodopa: Clinical features, mechanisms, and management strategies. Postgrad. Med. J..

[2] Melamed E. (1979). Early-morning dystonia. A late side effect of long-term levodopa therapy in Parkinson’s disease. Arch. Neurol..

[3] Markham C.H., Diamond S.G. (1986). Long-term follow-up of early dopa treatment in Parkinson’s disease. Ann. Neurol..

[4] Herin M., Colin M., Tursch B. (1976). Chemical Studies of Marine-Invertebrates. 25. Bull. Soc. Chim. Belg..

[5] Lin Y.S., Chen C.H., Liaw C.C., Chen Y.C., Kuo Y.H., Shen Y.C. (2009). Cembrane diterpenoids from the Taiwanese soft coral Sinularia flexibilis. Tetrahedron.

[6] Liu C.I., Chen C.C., Chen J.C., Su J.H., Huang H.H., Chen J.Y., Wu Y.J. (2011). Proteomic analysis of anti-tumor effects of 11-dehydrosinulariolide on CAL-27 cells. Mar. Drugs.

[7] Su T.R., Tsai F.J., Lin J.J., Huang H.H., Chiu C.C., Su J.H., Yang Y.T., Chen J.Y., Wong B.S., Wu Y.J. (2012). Induction of apoptosis by 11-dehydrosinulariolide via mitochondrial dysregulation and ER stress pathways in human melanoma cells. Mar. Drugs.

[8] Chen W.F., Chakraborty C., Sung C.S., Feng C.W., Jean Y.H., Lin Y.Y., Hung H.C., Huang T.Y., Huang S.Y., Su T.M. (2012). Neuroprotection by marine-derived compound, 11-dehydrosinulariolide, in an in vitro Parkinson’s model: A promising candidate for the treatment of Parkinson’s disease. Naunyn-Schmiedebergs Arch. Pharmacol..

[9] Bonifati V., Rizzu P., van Baren M.J., Schaap O., Breedveld G.J., Krieger E., Dekker M.C., Squitieri F., Ibanez P., Joosse M. (2003). Mutations in the DJ-1 gene associated with autosomal recessive early-onset parkinsonism. Science.

[10] Abou-Sleiman P.M., Healy D.G., Quinn N., Lees A.J., Wood N.W. (2003). The role of pathogenic DJ-1 mutations in Parkinson’s disease. Ann. Neurol..

[11] Healy D.G., Abou-Sleiman P.M., Valente E.M., Gilks W.P., Bhatia K., Quinn N., Lees A.J., Wood N.W. (2004). DJ-1 mutations in Parkinson’s disease. J. Neurol. Neurosurg. Psychiatry.

[12] Van Duijn C.M., Dekker M.C., Bonifati V., Galjaard R.J., Houwing-Duistermaat J.J., Snijders P.J., Testers L., Breedveld G.J., Horstink M., Sandkuijl L.A. (2001). Park7, a novel locus for autosomal recessive early-onset parkinsonism, on chromosome 1p36. Am. J. Hum. Genet..

[13] Chen P.C., Vargas M.R., Pani A.K., Smeyne R.J., Johnson D.A., Kan Y.W., Johnson J.A. (2009). Nrf2-mediated neuroprotection in the MPTP mouse model of Parkinson’s disease: Critical role for the astrocyte. Proc. Natl. Acad. Sci. USA.

[14] Inden M., Taira T., Kitamura Y., Yanagida T., Tsuchiya D., Takata K., Yanagisawa D., Nishimura K., Taniguchi T., Kiso Y. (2006). PARK7 DJ-1 protects against degeneration of nigral dopaminergic neurons in Parkinson’s disease rat model. Neurobiol. Dis..

[15] Gao H., Yang W., Qi Z., Lu L., Duan C., Zhao C., Yang H. (2012). DJ-1 protects dopaminergic neurons against rotenone-induced apoptosis by enhancing ERK-dependent mitophagy. J. Mol. Biol..

[16] Clements C.M., McNally R.S., Conti B.J., Mak T.W., Ting J.P. (2006). DJ-1, a cancer- and Parkinson’s disease-associated protein, stabilizes the antioxidant transcriptional master regulator Nrf2. Proc. Natl. Acad. Sci. USA.

[17] Huang H.C., Nguyen T., Pickett C.B. (2000). Regulation of the antioxidant response element by protein kinase C-mediated phosphorylation of NF-E2-related factor 2. Proc. Natl. Acad. Sci. USA.

[18] Barone M.C., Bohmann D. (2013). Assessing neurodegenerative phenotypes in *Drosophila* dopaminergic neurons by climbing assays and whole brain immunostaining. J. Vis. Exp..

[19] Stahl K., Mylonakou M.N., Skare O., Amiry-Moghaddam M., Torp R. (2011). Cytoprotective effects of growth factors: BDNF more potent than GDNF in an organotypic culture model of Parkinson’s disease. Brain Res..

[20] Allen S.J., Watson J.J., Shoemark D.K., Barua N.U., Patel N.K. (2013). GDNF, NGF and BDNF as therapeutic options for neurodegeneration. Pharmacol. Ther..

[21] Feng C.W., Wen Z.H., Huang S.Y., Hung H.C., Chen C.H., Yang S.N., Chen N.F., Wang H.M., Hsiao C.D., Chen W.F. (2014). Effects of 6-hydroxydopamine exposure on motor activity and biochemical expression in zebrafish (*Danio rerio*) larvae. Zebrafish.

[22] Junn E., Jang W.H., Zhao X., Jeong B.S., Mouradian M.M. (2009). Mitochondrial localization of DJ-1 leads to enhanced neuroprotection. J. Neurosci. Res..

[23] Zhang L., Shimoji M., Thomas B., Moore D.J., Yu S.W., Marupudi N.I., Torp R., Torgner I.A., Ottersen O.P., Dawson T.M. (2005). Mitochondrial localization of the Parkinson’s disease related protein DJ-1: Implications for pathogenesis. Hum. Mol. Genet..

[24] Im J.Y., Lee K.W., Junn E., Mouradian M.M. (2010). DJ-1 protects against oxidative damage by regulating the thioredoxin/ASK1 complex. Neurosci. Res..

[25] Kim S.J., Park Y.J., Hwang I.Y., Youdim M.B., Park K.S., Oh Y.J. (2012). Nuclear translocation of DJ-1 during oxidative stress-induced neuronal cell death. Free Radic. Biol. Med..

[26] Kim J.H., Choi D.J., Jeong H.K., Kim J., Kim D.W., Choi S.Y., Park S.M., Suh Y.H., Jou I., Joe E.H. (2013). DJ-1 facilitates the interaction between STAT1 and its phosphatase, SHP-1, in brain microglia and astrocytes: A novel anti-inflammatory function of DJ-1. Neurobiol. Dis..

[27] Birkmayer W., Riederer P., Ambrozi L., Youdim M.B. (1977). Implications of combined treatment with ‘Madopar’ and l-deprenil in Parkinson’s disease. A long-term study. Lancet.

[28] Xiao H., Lv F., Xu W., Zhang L., Jing P., Cao X. (2011). Deprenyl prevents MPP^+^-induced oxidative damage in PC12 cells by the upregulation of Nrf2-mediated NQO1 expression through the activation of PI3K/Akt and Erk. Toxicology.

[29] Nakaso K., Nakamura C., Sato H., Imamura K., Takeshima T., Nakashima K. (2006). Novel cytoprotective mechanism of anti-parkinsonian drug deprenyl: PI3K and Nrf2-derived induction of antioxidative proteins. Biochem. Biophys. Res. Commun..

[30] Wu R.M., Mohanakumar K.P., Murphy D.L., Chiueh C.C. (1994). Antioxidant mechanism and protection of nigral neurons against MPP^+^ toxicity by deprenyl (selegiline). Ann. N. Y. Acad. Sci..

[31] Youdim M.B., Tipton K.F. (2002). Rat striatal monoamine oxidase-B inhibition by l-deprenyl and rasagiline: Its relationship to 2-phenylethylamine-induced stereotypy and Parkinson’s disease. Parkinsonism Relat. Disord..

[32] Schroeter H., Bahia P., Spencer J.P., Sheppard O., Rattray M., Cadenas E., Rice-Evans C., Williams R.J. (2007). (-)Epicatechin stimulates ERK-dependent cyclic AMP response element activity and up-regulates GluR2 in cortical neurons. J. Neurochem..

[33] Joseph M.S., Ying Z., Zhuang Y., Zhong H., Wu A., Bhatia H.S., Cruz R., Tillakaratne N.J., Roy R.R., Edgerton V.R. (2012). Effects of diet and/or exercise in enhancing spinal cord sensorimotor learning. PLoS ONE.

[34] Jagatha B., Mythri R.B., Vali S., Bharath M.M. (2008). Curcumin treatment alleviates the effects of glutathione depletion in vitro and in vivo: Therapeutic implications for Parkinson’s disease explained via in silico studies. Free Radic. Biol. Med..

[35] Zbarsky V., Datla K.P., Parkar S., Rai D.K., Aruoma O.I., Dexter D.T. (2005). Neuroprotective properties of the natural phenolic antioxidants curcumin and naringenin but not quercetin and fisetin in a 6-OHDA model of Parkinson’s disease. Free Radic. Res..

[36] Vilotti S., Codrich M., Dal Ferro M., Pinto M., Ferrer I., Collavin L., Gustincich S., Zucchelli S. (2012). Parkinson’s disease DJ-1 L166P alters rRNA biogenesis by exclusion of TTRAP from the nucleolus and sequestration into cytoplasmic aggregates via TRAF6. PLoS ONE.

[37] Yang Y.C., Lii C.K., Lin A.H., Yeh Y.W., Yao H.T., Li C.C., Liu K.L., Chen H.W. (2011). Induction of glutathione synthesis and heme oxygenase 1 by the flavonoids butein and phloretin is mediated through the ERK/Nrf2 pathway and protects against oxidative stress. Free Radic. Biol. Med..

[38] Jean Y.H., Chen W.F., Duh C.Y., Huang S.Y., Hsu C.H., Lin C.S., Sung C.S., Chen I.M., Wen Z.H. (2008). Inducible nitric oxide synthase and cyclooxygenase-2 participate in anti-inflammatory and analgesic effects of the natural marine compound lemnalol from Formosan soft coral Lemnalia cervicorni. Eur. J. Pharmacol..

[39] Jean Y.H., Chen W.F., Sung C.S., Duh C.Y., Huang S.Y., Lin C.S., Tai M.H., Tzeng S.F., Wen Z.H. (2009). Capnellene, a natural marine compound derived from soft coral, attenuates chronic constriction injury-induced neuropathic pain in rats. Br. J. Pharmacol..

[40] Committee for the Update of the Guide for the Care and Use of Laboratory (2011). Animals Guide for the Care and Use of Laboratory Animals.

[41] Kilkenny C., Browne W.J., Cuthill I.C., Emerson M., Altman D.G. (2010). Improving bioscience research reporting: The ARRIVE guidelines for reporting animal research. J. Pharmacol. Pharmacother..

[42] McGrath J.C., Lilley E. (2015). Implementing guidelines on reporting research using animals (ARRIVE, etc.): New requirements for publication in BJP. Br. J. Pharmacol..

[43] Ungerstedt U. (1971). Striatal dopamine release after amphetamine or nerve degeneration revealed by rotational behaviour. Acta Physiol. Scand..

[44] Pycock C.J. (1980). Turning behaviour in animals. Neuroscience.

[45] Carman L.S., Gage F.H., Shults C.W. (1991). Partial lesion of the substantia nigra: Relation between extent of lesion and rotational behavior. Brain Res..

[46] Allbutt H.N., Henderson J.M. (2007). Use of the narrow beam test in the rat, 6-hydroxydopamine model of Parkinson’s disease. J. Neurosci. Methods.

